# Elevated brain aluminium and early onset Alzheimer’s disease in an individual occupationally exposed to aluminium: a case report

**DOI:** 10.1186/1752-1947-8-41

**Published:** 2014-02-10

**Authors:** Christopher Exley, Thomas Vickers

**Affiliations:** 1 The Birchall Centre, Lennard-Jones Laboratories, Keele University, Staffordshire, UK; 2 The Huxley Building, Life Sciences, Keele University, Staffordshire, UK

**Keywords:** Alzheimer’s disease, Brain aluminium, Occupational exposure to aluminium

## Abstract

**Introduction:**

Aluminium is a known neurotoxin and occupational exposure to aluminium has been implicated in neurological disease including Alzheimer’s disease. Here we present the first comprehensive and unequivocal data demonstrating significantly elevated brain aluminium content in an individual occupationally exposed to aluminium.

**Case presentation:**

A 66-year-old Caucasian man who died with Alzheimer’s disease showed significantly elevated brain aluminium content, 2.98 (2.73) μg/g dry weight, n = 46, following occupational exposure to aluminium over a period of 8 years.

**Conclusions:**

That the individual developed an early onset aggressive form of Alzheimer’s disease suggests a role for aluminium in disease aetiology. That the exposure to aluminium was through occupational exposure to aluminium dust suggests a prominent role for the olfactory system and lungs in the accumulation of aluminium in the brain.

## Introduction

Humans are experiencing a burgeoning exposure to aluminium in everyday life [1]. Aluminium accumulates in the brain with age [2] and exposure is associated with a number of neurodegenerative diseases [3]. Occupational exposure to aluminium has been linked with neurodevelopmental effects [4-6], although there are very limited data to describe brain aluminium content in occupationally exposed individuals [7]. Here we report the first data demonstrating significantly elevated content of brain aluminium in an individual diagnosed with Alzheimer’s disease following occupational exposure to aluminium.

## Case presentation

In 2003 a 58-year-old Caucasian man with no previous medical history of note was diagnosed with Alzheimer’s disease. Ten years previous to this he began to work with the preparation of a novel material (DARMATT KM1) used as insulation in the nuclear fuel and space industries. This work exposed him to aluminium sulphate ‘dust’ on a daily basis over 8 years. An ‘ordinary’ dust mask was supplied to protect against inhalation of the materials. Within a short time of starting this work he complained of headaches, tiredness and mouth ulcers. By 1999 he started to show problems in relation to memory and suffered depression. Following his death, aged 66, in 2011, at the request of the family and the local coroner, samples of his brain tissue were sent to the John Radcliffe Hospital, Oxford University, UK, for clinical diagnosis and a section of deep frozen frontal lobe was sent to Professor C Exley (Keele University, UK) for determination of tissue aluminium. Clinical diagnosis reported an abundance of argyrophilic β amyloid plaques and a profusion of neurofibrillary tangles in all areas of his cerebral cortex; the neuropathology is consistent with features of advanced Alzheimer’s disease.

The frontal lobe tissue (approximately 20g frozen weight) was allowed to thaw and then divided up into 50 similar-sized portions each weighing approximately 300mg. These were placed in an incubator at 37°C and allowed to achieve a constant dry weight over approximately 72 hours (Table 1). The dry tissues were then digested using a 1:1 mixture of 15.8M nitric acid and 30% w/v hydrogen peroxide in a microwave oven using established methods [8]. All samples produced clear digests and the total aluminium in each was measured by transversely heated graphite furnace atomic absorption spectrometry using established and fully verified methods [8]. The aluminium contents of three tissue samples (identification [IDs] 6, 8 and 9) were below the value of the method blank and were recorded as ‘zero’ for statistical purposes. The dry weights of three tissue samples (IDs 2, 14 and 18) were below 10mg and the aluminium contents of these samples were also excluded from statistical analyses because such very low dry weights can disproportionately influence the final aluminium content [8]. The mean aluminium content of the remaining tissues (n = 46) was 2.98 with a standard deviation of 2.73μg/g dry weight and a range from 0.00 to 12.97μg/g dry weight (Table 1).

**Table 1:** Aluminium content of 49 tissue samples taken from the frontal lobe of an individual with Alzheimer’s disease

**Brain sample ID**	**Wet weight (g)**	**Dry weight (g)**	**Weight change (%)**	**Al μg/g dry weight**
1	0.431	0.044	89.8	7.59
2*	0.167	0.002	98.8	33.11
3	0.263	0.017	93.5	2.06
4	0.243	0.022	90.9	1.35
5	0.260	0.019	92.7	4.62
6	0.234	0.014	94.0	<MB
7	0.192	0.026	86.5	2.54
8	0.165	0.016	90.4	<MB
9	0.153	0.020	87.0	<MB
10	0.173	0.012	93.1	1.96
11	0.242	0.013	94.6	2.22
12	0.290	0.090	69.0	0.92
13	0.229	0.026	88.6	2.26
14*	0.208	0.006	97.1	54.07
15	0.384	0.030	92.2	4.41
16	0.250	0.017	93.2	2.58
17	0.362	0.027	92.5	3.42
18*	0.219	0.001	99.5	211.67
19	0.401	0.025	93.8	12.97
20	0.301	0.028	90.7	4.16
21	0.217	0.016	92.6	2.26
22	0.405	0.033	91.8	4.02
23	0.419	0.045	89.3	4.81
24	0.513	0.107	79.1	0.93
25	0.457	0.028	93.9	12.34
26	0.516	0.069	86.6	1.60
27	0.359	0.039	89.1	1.76
28	0.294	0.023	92.2	3.84
29	0.321	0.024	92.3	8.55
30	0.435	0.060	86.2	2.33
31	0.284	0.029	89.8	3.79
32	0.450	0.042	90.7	1.47
33	0.543	0.061	88.8	1.85
34	0.388	0.042	89.2	2.45
35	0.604	0.092	84.8	1.02
36	0.392	0.071	81.9	1.04
37	0.422	0.116	72.5	0.61
38	0.288	0.036	87.5	5.45
39	0.611	0.236	61.4	1.39
40	0.353	0.081	77.0	3.24
41	0.394	0.106	73.1	2.35
42	0.395	0.100	74.7	2.60
43	0.479	0.161	66.4	1.89
44	0.492	0.171	65.2	1.03
45	0.423	0.126	70.2	1.31
46	0.309	0.059	80.9	0.89
47	0.382	0.098	74.3	3.57
48	0.318	0.076	76.1	2.72
49	0.267	0.055	79.4	2.70
**Mean (SD)**	**0.354 (0.113)**	**0.058 (0.048)**	**85.0 (9.1)**	**2.98 (2.73)**

Al, aluminium; MB, method blank; SD, standard deviation; *Excluded from mean.

## Conclusions

It is extremely rare to be given approximately 20g of brain tissue for elemental analysis. This opportunity enabled the most thorough analysis of the aluminium content of a single brain region from one individual ever undertaken. The data are revealing in respect of the wide range of aluminium contents recorded, confirming the suspected focal accumulation of aluminium in human brain tissue, and in respect of a mean value for 46 samples, 2.98μg/g dry weight, which is more than three times higher than a mean value, 0.83μg/g dry weight, previously recorded for multiple samples of frontal lobe from multiple individuals [8]. Excluding the three very high values (IDs 2, 14 and 18) 30% of the aluminium contents measured were higher than 3.50μg/g dry weight and could be considered potentially pathological [3,8]. The opportunity to analyse up to 50 separate tissue samples from one brain region has provided unequivocal evidence of an excessive load of aluminium in the frontal lobe of an individual who was occupationally exposed to aluminium over a period of 8 years. The clinical diagnosis of early onset sporadic Alzheimer’s disease showing features postmortem of advanced disease at age 66 is suggestive of aggressive disease aetiology and the probable involvement of aluminium in the onset and progression of the condition. High brain tissue aluminium was similarly implicated in a recent case of congophilic amyloid angiopathy where disease onset was again very early and disease pathology postmortem was highly advanced in an individual in their late 50s [9]. While it is impossible to know if high levels of brain aluminium instigated disease in either of these cases it is highly likely, considering the known neurotoxicity of aluminium, that aluminium was a contributor to disease aetiology, perhaps resulting in an earlier onset and more rapid progression of a nascent condition.

## Consent

Written informed consent was obtained from the patient’s next of kin for publication of this case report. A copy of the written consent is available for review by the Editor-in-Chief of this journal.

## Competing interests

The authors declare that they have no competing interests.

## Authors’ contributions

CE designed the study and participated in the measurements of aluminium. TV performed the digests of brain tissue and participated in the measurements of aluminium. CE wrote the manuscript. TV and CE agreed with the final version of the manuscript. Both authors read and approved the final manuscript.
